# Association between Density of Coronary Artery Calcification and Serum Magnesium Levels among Patients with Chronic Kidney Disease

**DOI:** 10.1371/journal.pone.0163673

**Published:** 2016-09-23

**Authors:** Yusuke Sakaguchi, Takayuki Hamano, Chikako Nakano, Yoshitsugu Obi, Isao Matsui, Yasuo Kusunoki, Daisuke Mori, Tatsufumi Oka, Nobuhiro Hashimoto, Yoshitsugu Takabatake, Atsushi Takahashi, Jun-Ya Kaimori, Toshiki Moriyama, Ryohei Yamamoto, Masaru Horio, Ken Sugimoto, Koichi Yamamoto, Hiromi Rakugi, Yoshitaka Isaka

**Affiliations:** 1 Department of Comprehensive Kidney Disease Research, Osaka University Graduate School of Medicine, 2-2 Yamada-oka, Suita 565-0871, Japan; 2 Department of Internal Medicine, Kisei Hospital, 1-18-4 Nishi-mikuni, Yodogawa-ku, Osaka 532-0006, Japan; 3 Department of Nephrology, Osaka University Graduate School of Medicine, 2-2 Yamada-oka, Suita 565-0871, Japan; 4 Department of Advanced Technology for Transplantation, Osaka University Graduate School of Medicine, 2-2 J8 Yamada-oka, Suita 565-0871, Japan; 5 Health Care Center, Osaka University, 1-17 Machikaneyama-cho, Toyonaka 560-0043, Japan; 6 Department of Functional Diagnostic Science, Osaka University Graduate School of Medicine, 2-2 Yamada-oka, Suita 565-0871, Japan; 7 Department of Geriatric and General Medicine, Osaka University Graduate School of Medicine, 2-2 Yamada-oka, Suita 565-0871, Japan; The University of Tokyo, JAPAN

## Abstract

**Background:**

The Agatston score, commonly used to quantify coronary artery calcification (CAC), is determined by the plaque area and density. Despite an excellent predictability of the Agatston score for cardiovascular events, the density of CAC has never been studied in patients with pre-dialysis chronic kidney disease (CKD). This study aimed to analyze the CAC density and its association with serum mineral levels in CKD.

**Methods:**

We enrolled patients with pre-dialysis CKD who had diabetes mellitus, prior cardiovascular disease history, elevated low-density lipoprotein cholesterol levels, or smoking history. The average CAC density was calculated by dividing the Agatston score by the total area of CAC.

**Results:**

The mean estimated glomerular filtration rate (eGFR) of 109 enrolled patients was 35.7 mL/min/1.73 m^2^. The correlation of the Agatston score with density was much weaker than that with the total area (R^2^ = 0.19, *P* < 0.001; and R^2^ = 0.99, *P* < 0.001, respectively). Multivariate analyses showed that serum magnesium level was inversely associated with the density, but not with the total area, after adjustment for demographics and clinical factors related to malnutrition-inflammation-atherosclerosis syndrome and mineral and bone disorders including fibroblast growth factor 23 (*P* = 0.006). This inverse association was pronounced among patients with higher serum phosphate levels (*P* for interaction = 0.02).

**Conclusion:**

CAC density was inversely associated with serum magnesium levels, particularly in patients with higher serum phosphate levels.

## Introduction

The Agatston method is the most widely used scoring system for the evaluation of coronary artery calcification (CAC). Its remarkable predictability for cardiac events is well established not only in the general population [1, 2] but also in patients with chronic kidney disease (CKD) [3–9]. The Agatston score is calculated from the product of a within-slice calcified plaque area and a plaque-specific density factor determined by computed tomography (CT) attenuation values, summed for all cardiac CT slices [10]. Consequently, the Agatston score becomes higher as plaque density increases. It is not evident, however, whether cardiovascular risk is actually augmented as the density increases. Notably, a recent report from a population-based cohort found that the lower CAC density was associated with a higher risk of coronary artery disease [11]. Another group also reported that CT attenuation values of calcified plaques were lower in patients with acute coronary syndrome than in those with stable angina [12]. These observations may be in line with a fact that a low-density spotty calcification is a typical feature of vulnerable plaques [13].

Applicability of these findings to patients with CKD, however, is uncertain because morphological characteristics of vascular calcification are quite different between patients with and without CKD. Autopsy studies revealed that the prevalence of dense calcification in both the tunica intima and media of the coronary arteries increases dramatically as kidney function declines [14–16]. It has also been reported that patients with CKD are more likely to have dense calcification at the culprit lesion of acute coronary syndrome than those without CKD [17]. Among several known factors that promote vascular calcification in patients with CKD, mineral disorders play a crucial role [18]; phosphate and calcium are the two major contributors to the development of medial calcification, whereas recent experimental studies have found the importance of magnesium as an anti-calcification mineral [19–26].

Given the unique features of vascular calcification in CKD, it is reasonable to assume that clinical implication of the CAC density is different between those with and without CKD. Since no study has examined the CAC density in pre-dialysis CKD, this study aimed to elucidate how CKD and its consequence of mineral disorders influence the CAC density in this population.

## Materials and Methods

### Study Setting

We recruited patients with pre-dialysis CKD who had diabetes mellitus, cardiovascular disease history, hyper-low-density lipoprotein cholesterolemia, or smoking history from the outpatient unit of Osaka University Hospital, Japan, from January 2013 to September 2015. These patients were part of the participants of our ongoing randomized clinical trial, which compares the efficacy of an orally administered carbon adsorbent (AST-120) and/or magnesium oxide on the prevention of CAC progression (UMIN000009738). All clinical information described in the current study were baseline data of this randomized trial and were collected before randomization. Diabetes mellitus was defined as 1) a fasting serum glucose level of ≥ 126 mg/dL and/or 2) a random serum glucose level of ≥ 200 mg/dL and/or 3) a hemoglobin A1c level of ≥ 6.5% and/or 4) prescription of glucose-lowering drugs including insulin therapy, and/or 5) presence of diabetic retinopathy. Prior history of cardiovascular disease included coronary artery disease, heart failure, cerebral infarction, cerebral hemorrhage, and peripheral artery disease. Hyper-low-density lipoprotein cholesterolemia was defined as 1) a serum low-density lipoprotein cholesterol level of ≥ 140 mg/dL and/or 2) prescription of statins. Patients were excluded if they were younger than 18 years, prescribed with magnesium-containing laxatives, or had previously undergone percutaneous coronary interventions.

The Ethics Committee of Osaka University Hospital approved the study protocol (Approval no. 12172–6). Written informed consent was obtained from all patients.

### Data Collection

Patients’ demographic and clinical characteristics including age, sex, height, body weight, systolic and diastolic blood pressure, heart rate, smoking history, medications, and medical history were collected by clinical research coordinators.

Blood samples were collected at study entry to measure the following parameters: albumin, creatinine, calcium, phosphate, magnesium, low-density lipoprotein cholesterol, C-reactive protein (CRP), hemoglobin, 1,25-dihydroxyvitamin D3 (double antibody radioimmunoassay assay), and whole parathyroid hormone (PTH) (immunoradiometric assay). Estimated glomerular filtration rate (eGFR) was calculated from the equation for the Japanese population [27]. CKD stages were defined as follows; CKD stage G3a (eGFR; 60 >, ≥ 45 mL/min/1.73m^2^), G3b (eGFR; 45 >, ≥ 30 mL/min/1.73m^2^), and G4 (eGFR; 30 >, ≥ 15 mL/min/1.73m^2^) [28]. Serum calcium level was adjusted as follows if serum albumin level was <4.0 g/dL: corrected serum calcium level (mg/dL) = measured serum calcium level (mg/dL)+(4.0 –serum albumin level (g/dL)).

We further measured serum intact fibroblast growth factor 23 (FGF23) levels (chemiluminescent enzyme immune assay, CL-JACK^®^; Kyowa Medics, Tokyo, Japan) using stored serum samples frozen at −80°C.

### Evaluation of CAC

CT scans were performed using Aqualon 16-slice multi-detector-row CT scanner (GE Healthcare Japan, Tokyo, Japan) with the following protocol: retrospective electrocardiogram gating, 120-kV tube voltage, 180-mAs tube current, 330-ms rotation time, 1.2 × 16-mm detector collimation, 3.0-mm reconstructed slice thickness (original slice thickness, 2.5 mm), and 0.2 pitch. CT images were transferred to SmartScore version 3.5 (GE Healthcare, Japan) wherein all the following analyses were performed. Calcification was defined as a minimum of two adjacent pixels with density more than 130 HU. The CAC was quantified by the Agatston score, which is calculated by multiplying the area of each calcified plaque by a density factor determined by a peak pixel intensity within the plaque, and these plaque-specific scores are added for all slices (10). The density factor is 1, 2, 3, and 4 for a plaque with peak intensities of 130 to 199 HU, 200 to 299 HU, 300 to 399 HU, and 400 HU or greater, respectively.

Based on the methodology used by Criqui et al. [11], the average CAC density was calculated using the volume score which is the product of the calcified area and the CT slice thickness. First, the total calcified area of each patient was obtained by dividing the volume score by the CT-slice thickness. Subsequently, the Agatston score was divided by the total calcified area; a resultant quotient reflects the average CAC density.

### Statistical Analysis

Patient characteristics were compared across CKD stages by using Cuzick trend test for continuous variables and Cochran-Armitage test for categorical variables. Correlation of the Agatston score with the area and density of the CAC was analyzed by Pearson product-moment correlation coefficient.

Multivariate associations of the Agatston score, area, and density with clinical factors were analyzed using a linear regression with robust standard errors due to a non-normality and heteroscedasticity of regression residuals. Dependent variables (Agatston score, area, and density) were transformed to accommodate skewed distribution. The Agatston score and the area were log-transformed after adding 1 to each value. The density was exponentially transformed. Relationship between serum magnesium level and density was illustrated by a restricted cubic spline curve with three knots. We tested a pre-specified interaction (serum magnesium vs. serum phosphate) by incorporating their product term in the original multivariate model. Subsequent subgroup analyses were performed by dividing patients at the median levels of serum phosphate. In this subgroup analysis, we included only covariates of a *P*-value less than 0.4 in the original multivariate model due to the limited number of samples in each subgroup. Because some data were missing on FGF23 and CRP, which were considered to occur completely at random, we performed a multiple imputation method with five data sets (a multivariate normal model).

All reported *P*-values were two-sided. A *P*-value < 0.05 was considered statistically significant. Statistical analyses were performed using StataIC 13 Statistical Software (StataCorp LP, College Station, TX).

## Results

**Fig 1:**
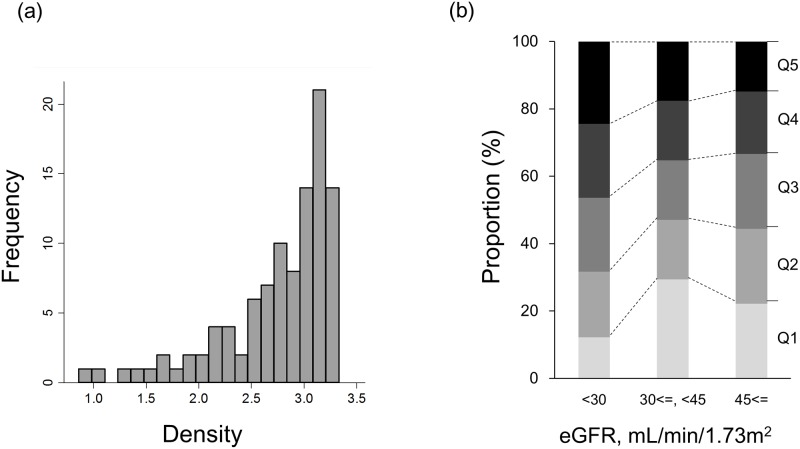
Distribution of the average density of coronary artery calcification in patients with chronic kidney disease. (a) histogram and (b) stacked bar charts according to CKD stages. Q1–Q5 denotes the 1st to 5th quintiles of the average density of the coronary artery calcification. Range of quintiles for the density: Q1, 0.86–2.36; Q2, 2.38–2.79; Q3, 2.81–3.04; Q4, 3.05–3.18; Q5, 3.18–3.33.

A total of 109 patients with CKD were enrolled. Patient characteristics according to CKD stages are shown in Table 1. There were no missing values in these data except for CRP which missed in 1 patient. The mean eGFR of all patients was 35.7 mL/min/1.73 m^2^. Nearly 80% of patients had diabetes mellitus. Distribution of the Agatston score and the total area of CAC was strongly right skewed (S1 Fig). The median Agatston score was 273, and 44% of patients had severe CAC (the Agatston score more than 400). Both the Agatston score and the total area of CAC became higher as the CKD stages advanced (Table 1, S1 Fig). The average CAC density was analyzed in 102 patients whose Agatston score was greater than 0. Distribution of the density was left skewed (Fig 1). The density also tended to be higher in the advanced stages of CKD, although the difference did not reach statistical significance (*P* = 0.09) (Table 1, Fig 1).

**Table 1:** Demographic and clinical characteristics of study participants.

		estimated glomerular filtration rate (mL/min/1.73m^2^)			
	Total	≥ 45	45 >, ≥ 30	30 >	
	n = 109	n = 29	n = 35	n = 45	*P* for trend
age, year	69.3 (11.7)	66.6 (11.7)	68.3 (12.4)	71.8 (10.9)	0.06
male, n (%)	84 (77.1)	22 (75.9)	28 (80.0)	34 (75.6)	0.9
body mass index, kg/m^2^	24.0 (4.0)	24.4 (4.6)	23.4 (4.2)	24.1 (3.4)	0.9
diabetes mellitus, n (%)	88 (80.7)	23 (79.3)	28 (80.0)	37 (82.2)	0.7
prior history of CVD, n (%)	44 (40.4)	7 (24.1)	13 (37.1)	24 (53.3)	0.01
systolic blood pressure, mmHg	132.9 (18.6)	135.1 (22.1)	132.1 (16.0)	132.0 (18.3)	0.5
diastolic blood pressure, mmHg	72.1 (12.1)	77.1 (11.7)	71.5 (12.2)	69.4 (11.6)	0.03
smoker, n(%)	14 (12.8)	4 (13.8)	4 (11.4)	6 (13.3)	0.9
eGFR, mL/min/1.73m^2^	35.7 (13.7)	54.2 (7.0)	37.0 (4.3)	22.8 (4.5)	<0.001
adj.Ca, mg/dL	9.25 (0.40)	9.34 (0.38)	9.27 (0.38)	9.17 (0.44)	0.07
phosphate, mg/dL	3.40 (0.60)	3.26 (0.55)	3.38 (0.68)	3.52 (0.55)	0.05
magnesium, mg/dL	2.06 (0.24)	1.96 (0.16)	2.06 (0.21)	2.12 (0.29)	0.006
C-reactive protein, mg/dL	0.06 [0, 0.20]	0.06 [0, 0.13]	0.06 [0, 0.28]	0.07 [0, 0.19]	0.3
hemoglobin, g/dL	12.6 (1.7)	13.6 (1.9)	12.6 (1.5)	11.9 (1.6)	<0.001
LDL-cholesterol, mg/dL	104 (30)	103 (30)	105 (38)	103 (24)	0.9
hemoglobin A1c, %	6.7 (1.0)	6.5 (0.8)	6.9 (1.1)	6.6 (1.1)	0.8
albumin, g/dL	4.0 (0.5)	4.2 (0.4)	4.0 (0.5)	3.9 (0.5)	0.01
1,25-dihydroxyvitaminD, pg/mL	38.5 (14.6)	45.8 (18.4)	37.6 (11.5)	34.6 (12.3)	0.006
whole PTH, pg/mL	47 [34, 75]	37 [28, 48]	41 [29, 55]	68 [51, 94]	<0.001
Agatston score	273 [72, 746]	139 [50, 454]	204 [61, 686]	570 [114, 1023]	0.007
area of CAC	105 [34, 252]	56 [21, 143]	71 [29, 252]	198 [42, 348]	0.009
density of CAC	2.91 [2.55, 3.15]	2.82 [2.58–3.11]	2.90 [2.25–3.16]	3.02 [2.67–3.16]	0.09

Data presented as number (percent), mean (standard deviation), or median [interquartile range].

Abbreviations: CKD, chronic kidney disease; CVD, cardiovascular disease; eGFR, estimated glomerular filtration rate; adj.Ca, adjusted calcium; LDL, low-density lipoprotein; PTH, parathyroid hormone; CAC, coronary artery calcification.

**Fig 2:**
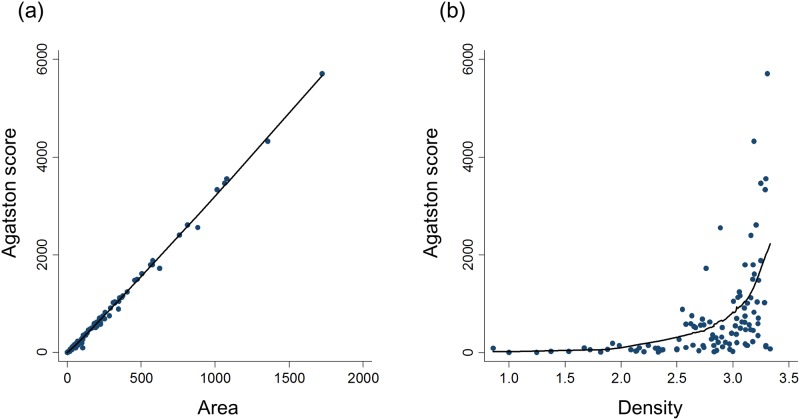
Scatterplots of the Agatston score versus (a) total area and (b) density of coronary artery calcification. R^2^ for linear regression is 0.99 (a) and 0.19 (b), respectively.

Scatterplots of the Agatston score versus the total area and density are displayed in Fig 2. The total area was closely correlated with the Agatston score (R^2^ = 0.99, *P* < 0.001), indicating that the Agatston score is determined mostly by the total area. In contrast, the relationship between density and the Agatston score was non-linear and not well fitted to a linear model (R^2^ = 0.19, *P* < 0.001).

**Fig 3:**
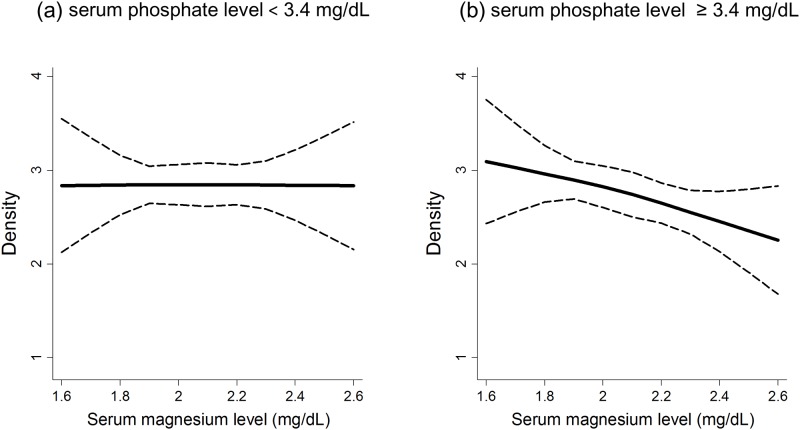
Cubic spline curves for the relationship between serum magnesium level and the coronary artery calcification density. (a) serum phosphate level < 3.4 mg/dL, (b) serum phosphate level ≥ 3.4 mg/dL. The model was adjusted for age, systolic blood pressure, estimated glomerular filtration rate, and adjusted serum calcium levels. The dashed line represents the 95% confidence interval.

Association between the log-transformed Agatston score and clinical characteristics is shown in S1 Table. Log-transformed Agatston score were significantly higher in patients with higher serum calcium level. Patients with older age and diabetes mellitus tended to have higher Agatston score. Similar results were obtained for the log-transformed total area of CAC (S2 Table). There were no significant interaction between serum magnesium and phosphate levels for both the Agatston score and the total area of CAC (*P* = 0.2 and 0.3, respectively). The exponential-transformed density of CAC was associated positively with age and negatively with serum magnesium levels (Table 2; model 1). These significant associations persisted after further adjustment for clinical factors related to malnutrition-inflammation-atherosclerosis (MIA) syndrome (serum albumin, CRP, and hemoglobin) (Table 2; model 2) and mineral and bone disorders (MBD) (1,25-dihydroxyvitamin D3 and whole PTH) (Table 2; model 3). We found a significant interaction between serum magnesium and phosphate levels for the CAC density in model 1 (*P* for interaction = 0.02). When patients were divided into two subgroups by the median serum phosphate level (3.4 mg/dL), the association between serum magnesium levels and density was only significant in the higher phosphate group (*P* = 0.001) but not in the lower phosphate group (*P* = 0.6) (Table 3, Fig 3). This interaction remained significant in model 2 (*P* for interaction = 0.03) and marginally significant in model 3 (*P* for interaction = 0.06).

**Table 2:** Multivariate associations between exponential-transformed density of CAC and clinical factors.

	Model 1		Model 2		Model 3	
*Covariates*	stand. β	P-value	stand. β	P-value	stand. β	P-value
age	0.45	<0.001	0.47	<0.001	0.49	<0.001
male	0.02	0.9	-0.02	0.9	0.002	0.9
body mass index	0.06	0.6	0.03	0.8	0.04	0.7
systolic blood pressure	-0.17	0.07	-0.15	0.1	-0.17	0.1
diabetes mellitus	-0.08	0.5	-0.04	0.7	-0.04	0.8
prior history of CVD	0.02	0.9	0.02	0.9	0.01	0.9
smoker	0.05	0.7	-0.01	0.9	-0.03	0.8
eGFR	-0.13	0.2	-0.21	0.07	-0.16	0.3
adj.Ca	0.12	0.3	0.12	0.3	0.16	0.1
phosphate	-0.08	0.4	-0.01	0.9	0.02	0.9
magnesium	-0.21	0.03	-0.23	0.01	-0.24	0.01
LDL-cholesterol	-0.02	0.9	-0.05	0.7	-0.07	0.5
albumin			0.06	0.6	0.07	0.6
C-reactive protein			-0.04	0.7	-0.06	0.6
hemoglobin			0.19	0.2	0.18	0.2
log(whole PTH)					0.15	0.3
1,25-dihydroxyvitaminD					0.01	0.9

Abbreviations: CAC, coronary artery calcification; CKD, chronic kidney disease; stand. β, standardized β-coefficient; CVD, cardiovascular disease; eGFR, estimated glomerular filtration rate; adj.Ca, adjusted calcium; LDL, low-density lipoprotein; PTH, parathyroid hormone.

Each Model includes all covariates listed in the first column.

**Table 3:** Subgroup analyses for the association between exponential-transformed density of CAC and clinical factors.

	serum phosphate <3.4 mg/dL		serum phosphate ≥3.4 mg/dL	
	n = 45		n = 57	
*Covariates*	stand. β	P-value	stand. β	P-value
age	0.33	0.02	0.48	0.002
systolic BP	-0.05	0.8	-0.12	0.3
eGFR	-0.26	0.07	-0.02	0.9
adj.Ca	0.25	0.07	0.02	0.8
magnesium	0.08	0.6	-0.36	0.001

Abbreviations: CAC, coronary artery calcification; stand. β, standardized β-coefficient; BP, blood pressure; eGFR, estimated glomerular filtration rate; adj.Ca, adjusted calcium

Models include all covariates listed in the first column.

Two additional analyses were performed to further investigate the relationship between magnesium and density. First, while the density was exponentially transformed due to its strongly skewed distribution, we still found a significant association between serum magnesium levels and density when the variable transformation was not performed (data not shown). Second, serum intact FGF23 levels were measured in 79 patients whose serum samples were stored. We used a multiple imputation method to impute the missing values for serum FGF23 levels as well as CRP. After including FGF23 into the model, the association between serum magnesium levels and density remained significant (*P* = 0.006; S3 Table). In this imputation model, the interaction between serum magnesium and phosphate levels remained significant (*P* for interaction = 0.04)

## Discussion

The Agatston score is the linear product of two parameters, the area and the density of CAC. Most of the previous studies have not evaluated these two variables separately. In this study, we computed the CAC density of pre-dialysis CKD patients and showed that while there was a good correlation between the Agatston score and the total area of CAC, the density was poorly correlated with the Agatston score. This result implies that the Agatston score is largely determined by the area whereas the information of the density score is not well reflected in the Agatston score. Therefore, despite a large body of evidence showing the relationship between the Agatston score and cardiovascular prognosis, clinical relevance of CAC density cannot be drawn from such studies.

In this context, the two previous studies of primarily the non-CKD population examined the CAC density [11, 12]. These studies found that the risk of coronary artery disease increased as the CAC density decreased, suggesting that high-density calcified plaques may be protective. In fact, it is believed that the early stage spotty calcification enhances plaque vulnerability [13]. However, the situation may be somewhat different in CKD patients who have high prevalence of dense calcification [14–17]. Dense calcification is considered to contribute to worse cardiovascular prognosis in a different manner from spotty calcification, for example, by increasing vascular stiffness and reducing coronary flow reserve [29]. In fact, contrary to the findings of the non-CKD population [11, 12], Bellasi et al. have recently reported that the higher CAC density was associated with an increased risk of all-cause mortality in a cohort of 125 prevalent hemodialysis patients [30], suggesting that clinical relevance of CAC density in patients with kidney disease is different from those without kidney disease. Further follow-up studies are needed to elucidate the relationship between CAC density and cardiovascular prognosis in non-dialysis CKD patients.

We found a significant association between the density and serum magnesium levels. This association was maintained after extensive adjustment for clinical factors related to MIA syndrome and MBD including FGF23. Recently, increasing evidence has shown anti-calcification property of magnesium and its protective effects on cardiovascular risks among patients with CKD [31–36]. In particular, several studies of dialysis patients found that lower serum magnesium levels are associated with higher frequency of calcifications of the peripheral arteries [37, 38]. However, the relationship between magnesium and vascular calcification, particularly CAC, among pre-dialysis CKD patients has never been investigated. To our knowledge, this study is the first to reveal that the higher serum magnesium levels are associated with lower CAC density in pre-dialysis CKD. In addition, there was a significant interaction between magnesium and phosphate levels, implying that the effect of magnesium on the density might be exerted particularly against the high phosphate condition. This is in line with in vitro and ex vivo studies showing an inhibitory effect of magnesium on phosphate-induced calcification of vascular smooth muscle cells [19–26].

In contrast to the strong relationship between the density and serum magnesium level, the area of CAC was not significantly associated with magnesium. Magnesium has been known to suppress the formation of calcium phosphate apatite and to stabilize amorphous calcium phosphate [39]. It is also known that whitlockite, a magnesium-substituted crystal, exists in human uremic arterial calcifications [40], although its clinical significance has not been clarified. We speculate that magnesium has higher effect on the chemical composition and structure of vascular calcification that would alter the CAC density, rather than the CAC volume. Further studies are required to elucidate how magnesium influences the crystalline feature of vascular calcification.

Several clinical factors are involved in the development of vascular calcification, among which aging has especially strong impact [41]. We confirmed that older age was associated with higher CAC density. On the other hand, despite a well-known role of phosphate in the process of vascular calcification, no significant association was found between serum phosphate levels and the density. This may be because the serum phosphate levels of our patients were not so high; thus, the influence of phosphate might be trivial. Several previous studies have also failed to find a significant association between serum phosphate levels and CAC [42, 43]. Finally, we could not find a significant association between the density and diabetes mellitus which is another important contributor to vascular calcification. This would be largely owing to the patient selection method. Because the density cannot be defined in those without CAC, we enrolled only patients with high risk of CAC; patients without diabetes mellitus had cardiovascular complications or some risk factors of vascular calcification. These criteria would make the effect of diabetes null. In addition, glycemic control of our patients was good. Therefore, the impact of diabetes may be underestimated in our study.

As previously mentioned by Criqui et al. [11], we have to acknowledge some limitations belonging to the computational methodology of the CAC density. First, this method only provides the average density of all calcified lesions but not the density of each lesion. Thus, it could be possible that low-density spotty calcifications coexist in patients having high-density score. Second, the density score is determined by the highest density pixel within each calcified lesion in the Agatston method. Therefore, the density of primarily low-density calcified plaque can be overestimated if it contains a small part of high-density pixels.

Other than the calculation methodology of the CAC density, some limitations should be noted in the interpretation of our findings. First, causal inference between magnesium and the density cannot be proven because of the observational study design, although numerous experimental studies have demonstrated inhibitory effects of magnesium on vascular calcification [19–26]. Second, the sample size was relatively small, hence we could include only a limited number of covariates in the multivariate models. Third, although we evaluated only serum magnesium levels as a marker of magnesium status, only 1% of the total body magnesium exists in the extracellular space. It has been suggested that intracellular magnesium levels are more sensitive marker for magnesium deficiency in the body [44]. Nevertheless, we could find the robust association between serum magnesium levels and the CAC density in this study which might imply that extracellular magnesium per se has some role in the pathophysiology of vascular calcification. Finally, this study recruited patients with a high risk of CAC. As a result, nearly 80% of patients had diabetes mellitus. Although this inclusion criterion is preferable from a clinical standpoint as vascular calcification is a much more important issue for diabetic patients, the generalizability of our findings to lower risk and non-diabetic population needs further confirmation.

In conclusion, we showed that the CAC density was not well correlated with the Agatston score; thus, it may have a distinct clinical implication especially for coronary events. Longitudinal studies should elucidate the cardiovascular prognostic value of the CAC density in CKD patients. Since there was a cross-sectional association between serum magnesium levels and the density, future interventional trials are warranted to elucidate the effects of magnesium on CAC.

## Supporting Information

S1 Fig Distributions of Agatston score and total area of coronary artery calcification in patients with chronic kidney disease. A histogram of (a) Agatston score and (b) total area of coronary artery calcification. Stacked bar charts of these parameters according to CKD stages are shown in (c) Agatston score and (d) total area. Q1–Q5 denotes the 1st to 5th quintiles of the total area of the coronary artery calcification. Range of quintiles for the total area: Q1, 0–21.2; Q2, 21.6–56.4; Q3, 59.6–177.6; Q4, 180.8–323.2; Q5, 347.6–1724.8.
(TIF)

S1 Table Multivariate association between log-transformed Agatston score and clinical characteristics of 109 CKD patients. (DOCX)

S2 Table Multivariate association between log-transformed total area of CAC and clinical factors of 109 CKD patients. (DOCX)

S3 Table Multiple imputation analysis for the association between exponential transformed density of CAC and clinical characteristics including serum FGF23. (DOCX)
