# Supplementary figures and images for: Association between Density of Coronary Artery Calcification and Serum Magnesium Levels among Patients with Chronic Kidney Disease
Source: PLoS One. 2016 Sep 23;11(9):e0163673. doi: 10.1371/journal.pone.0163673 (PMC5035086; doi:10.1371/journal.pone.0163673)

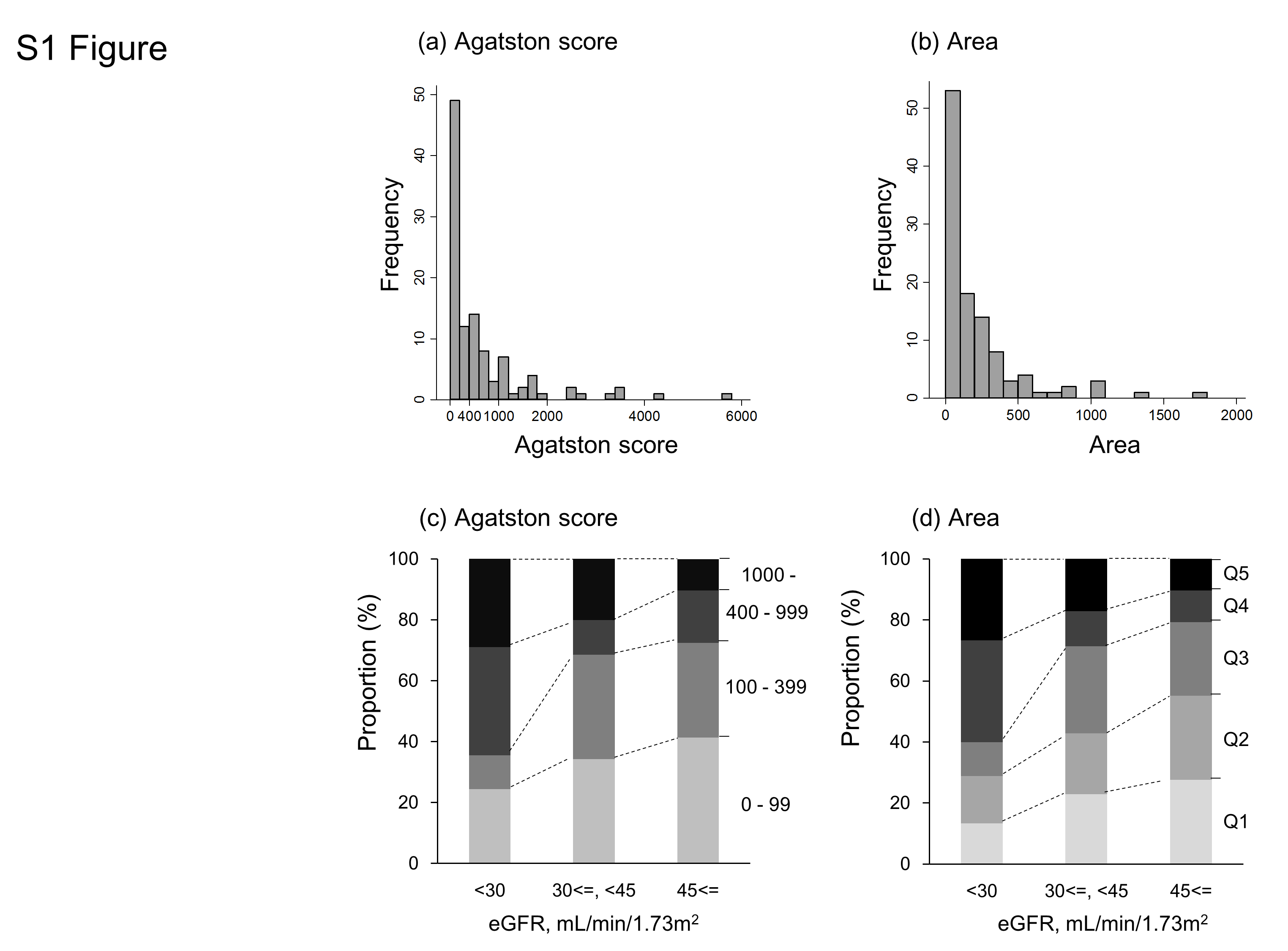

Supplement: S1 Fig — A histogram of (a) Agatston score and (b) total area of coronary artery calcification. Stacked bar charts of these parameters according to CKD stages are shown in (c) Agatston score and (d) total area. Q1–Q5 denotes the 1st to 5th quintiles of the total area of the coronary artery calcification. Range of quintiles for the total area: Q1, 0–21.2; Q2, 21.6–56.4; Q3, 59.6–177.6; Q4, 180.8–323.2; Q5, 347.6–1724.8. (TIF) [file pone.0163673.s001.tif]
