# Supplementary material for: Association between Density of Coronary Artery Calcification and Serum Magnesium Levels among Patients with Chronic Kidney Disease
Source: PLoS One. 2016 Sep 23;11(9):e0163673. doi: 10.1371/journal.pone.0163673 (PMC5035086; doi:10.1371/journal.pone.0163673)
Supplement: S2 Table — (DOCX) [file pone.0163673.s003.docx]

S2 Table. Multivariate association between log-transformed total area of CAC and clinical factors of 109 CKD patients

| *Covariates* | stand. β | P-value |
| --- | --- | --- |
| age | 0.22 | 0.07 |
| male | -0.05 | 0.6 |
| body mass index | 0.05 | 0.6 |
| systolic blood pressure | -0.07 | 0.4 |
| diabetes mellitus | 0.24 | 0.03 |
| prior history of CVD | 0.13 | 0.2 |
| smoker | 0.07 | 0.4 |
| eGFR | -0.14 | 0.2 |
| adj.Ca | 0.31 | 0.004 |
| phosphate | -0.08 | 0.4 |
| magnesium | 0.03 | 0.8 |
| LDL-cholesterol | -0.04 | 0.7 |

Abbreviations: CAC, coronary artery calcification; CKD, chronic kidney disease; stand. β, standardized β-coefficient; CVD, cardiovascular disease; eGFR, estimated glomerular filtration rate; adj.Ca, adjusted calcium; LDL, low-density lipoprotein

Model includes all covariates listed in the first column.
