# Supplementary material for: Association between Density of Coronary Artery Calcification and Serum Magnesium Levels among Patients with Chronic Kidney Disease
Source: PLoS One. 2016 Sep 23;11(9):e0163673. doi: 10.1371/journal.pone.0163673 (PMC5035086; doi:10.1371/journal.pone.0163673)
Supplement: S3 Table — (DOCX) [file pone.0163673.s004.docx]

S3 Table. Multiple imputation analysis for the association between exponential transformed density of CAC and clinical characteristics including serum FGF23

|  |  |  |  |
| --- | --- | --- | --- |
| *covariates* | β | Standard Error | P-value |
| age | 0.30 | 0.07 | <0.001 |
| male | 0.26 | 1.74 | 0.9 |
| body mass index | 0.03 | 0.20 | 0.9 |
| systolic BP | -0.05 | 0.04 | 0.2 |
| diabetes mellitus | -0.97 | 2.23 | 0.7 |
| prior history of CVD | -0.37 | 1.48 | 0.8 |
| smoker | 0.41 | 2.31 | 0.9 |
| eGFR | -0.10 | 0.08 | 0.2 |
| adj.Ca | 2.41 | 1.78 | 0.2 |
| phosphate | 0.50 | 1.44 | 0.7 |
| magnesium | -7.06 | 2.52 | 0.006 |
| LDL-cholesterol | -0.01 | 0.03 | 0.6 |
| albumin | 1.02 | 1.64 | 0.5 |
| log (whole PTH) | 1.13 | 1.67 | 0.5 |
| 1.25-dyhydroxyvitamin D | -0.02 | 0.07 | 0.7 |
| log FGF23 | -1.68 | 1.45 | 0.3 |

Abbreviations: CAC, coronary artery calcification; BP, blood pressure; CVD, cardiovascular disease; eGFR, estimated glomerular filtration rate; adj.Ca, adjusted calcium; LDL, low-density lipoprotein; PTH, parathyroid hormone; FGF23, fibroblast growth factor 23.

Model includes all covariates listed in the first column.
